# Use of Anthropogenic Sea Floor Structures by Australian Fur Seals: Potential Positive Ecological Impacts of Marine Industrial Development?

**DOI:** 10.1371/journal.pone.0130581

**Published:** 2015-07-01

**Authors:** John P. Y. Arnould, Jacquomo Monk, Daniel Ierodiaconou, Mark A. Hindell, Jayson Semmens, Andrew J. Hoskins, Daniel P. Costa, Kyler Abernathy, Greg J. Marshall

**Affiliations:** 1 School of Life and Environmental Sciences, Deakin University, Burwood, VIC, Australia; 2 Institute of Marine and Antarctic Science, University of Tasmania, Hobart, Tasmania; 3 Department of Ecology and Evolutionary Biology, Institute of Marine Sciences, UC Santa Cruz, Santa Cruz, California, United States of America; 4 Remote Imaging Department, National Geographic Society, Washington, D.C., United States of America; Sonoma State University, UNITED STATES

## Abstract

Human-induced changes to habitats can have deleterious effects on many species that occupy them. However, some species can adapt and even benefit from such modifications. Artificial reefs have long been used to provide habitat for invertebrate communities and promote local fish populations. With the increasing demand for energy resources within ocean systems, there has been an expansion of infrastructure in near-shore benthic environments which function as *de facto* artificial reefs. Little is known of their use by marine mammals. In this study, the influence of anthropogenic sea floor structures (pipelines, cable routes, wells and shipwrecks) on the foraging locations of 36 adult female Australian fur seals (*Arctocephalus pusillus doriferus*) was investigated. For 9 (25%) of the individuals, distance to anthropogenic sea floor structures was the most important factor in determining the location of intensive foraging activity. Whereas the influence of anthropogenic sea floor structures on foraging locations was not related to age and mass, it was positively related to flipper length/standard length (a factor which can affect manoeuvrability). A total of 26 (72%) individuals tracked with GPS were recorded spending time in the vicinity of structures (from <1% to >75% of the foraging trip duration) with pipelines and cable routes being the most frequented. No relationships were found between the amount of time spent frequenting anthropogenic structures and individual characteristics. More than a third (35%) of animals foraging near anthropogenic sea floor structures visited more than one type of structure. These results further highlight potentially beneficial ecological outcomes of marine industrial development.

## Introduction

Anthropogenic alterations to natural habitats can often have deleterious effects on species occurring within them [1]. Changes in land-use and sea-use can lead to a reduction in foraging habitat, breeding sites and refuge from predators for many species [2–5]. Some species, however, can adapt to, and even benefit from, habitat modifications. Indeed, anthropogenic structures erected as a consequence of such changes provide a range of benefits for some species from predator avoidance, thermoregulation, and breeding sites to acting as foraging areas or facilities to improve foraging [6–8].

Artificial reefs are anthropogenic structures deposited or constructed on an otherwise featureless sea floor that promote marine life. The structures provide a substrate for epifaunal life, the increased vertical habitat heterogeneity promotes the biodiversity of invertebrates and their structural complexity affords shelter for small fish and cephalopods [9]. As the biomass of epifaunal species and those that feed on them increases, larger predatory fish are attracted to these sites [10]. Consequently, artificial reefs have been used extensively around the world to increase the local density of fish for recreational and commercial fishermen [11, 12]. In addition, marine industrial structures (e.g. oil rigs, pipelines) have been shown to develop into important habitats for sessile invertebrates and fish communities, leading to calls for them to be converted in artificial reefs once they become obsolete [13–15]. However, the use of such artificial reefs by marine mammals has, until recently, received little attention and their potential importance as foraging zones has been investigated in few species [16–18].

Pinnipeds (seals, sea lions and walruses) around the world have experienced divergent rates of population recovery since the end of the commercial sealing era [19]. Whereas pelagic feeding species have experienced rapid growth in numbers, populations of benthic foraging species have increased very slowly, are stable or in decline [20]. It has been suggested that the low population recovery rates of benthic species could be due to them working at or near their physiological limit [21] hunting cryptic prey in continental shelf environments which for decades have been the focus of commercial fisheries employing bottom trawlers that disrupt the habitat and remove the larger size-classes of species that the seals depend on [22, 23].

**Fig 1:**
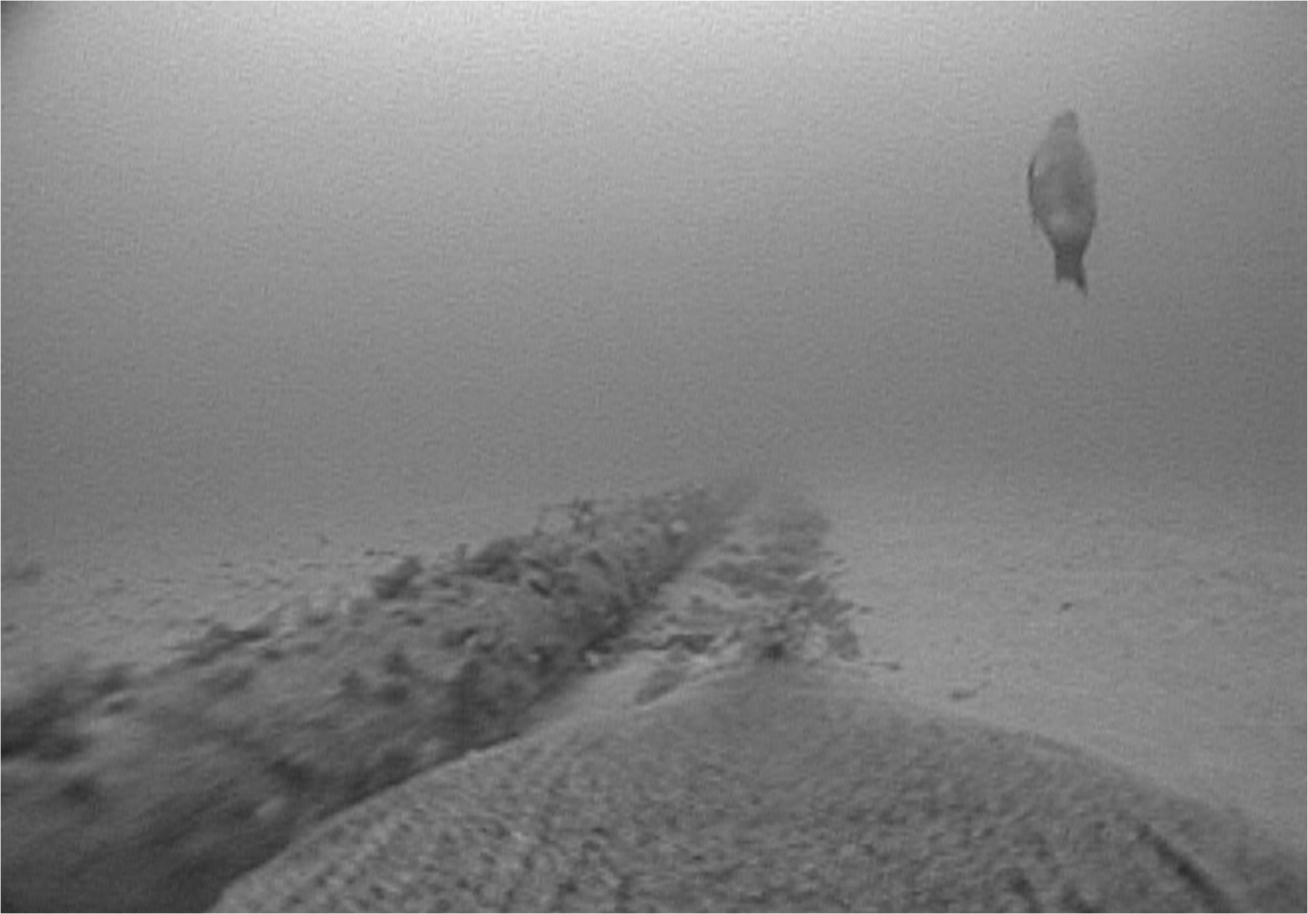
Image taken by animal-borne video camera on a female Australian fur seal foraging along a gas pipeline showing the sessile invertebrates and another fur seal.

The Australian fur seal (*Arctocephalus pusillus doriferus*) is one such benthic foraging species, feeding exclusively over the continental shelf on a wide variety of demersal fish and cephalopod species [24, 25]. While its population (*ca*120000 individuals) is slowly recovering from near-extinction after the commercial sealing era of the 18^th^ and 19^th^ centuries [26], it is still currently at <60% of its estimated pre-exploitation level [27]. All but one of its 13 breeding colonies occur on islands within Bass Strait [27], the shallow continental shelf region between the Australian mainland and Tasmania which has a relatively uniform bathymetry (average depth 60 m), few features and is considered to be a region of low primary productivity [28]. Therefore, the anthropogenic structures (e.g. oil/gas rigs, pipelines) that occur on its relatively featureless sea floor could provide valuable prey habitat and promote foraging success for the species. Indeed, recent data from animal-borne video cameras revealed individuals hunting near pipelines and oil rigs (Fig 1). Association with such *de-facto* artificial reefs could have important implications for the species’ recovery, its response to environmental variability and the potential impacts of further industrial developments within its foraging range. It is not known, however, to what extent Australian fur seals use such areas as foraging sites.

The aims of this study, therefore, were to determine in female Australian fur seals: 1) whether foraging patterns are influenced by anthropogenic sea floor structures; 2) the proportion of time spent in association with such structures; and 3) whether proxies of animal age, foraging experience, and manoeuvrability influence foraging in association with anthropogenic structures.

## Material and Methods

### Ethics statement

All work was carried out with approval of the Deakin University Animal Ethics Committee (A16-2008) and under Department of Sustainability and Environment (Victoria, Australia) Wildlife Research Permit (10005848). Kanowna Island is part of the Wilsons Promontory Marine National Park and was accessed under permit from Parks Victoria.

### Animal handling and instrumentation

The study was conducted at the Australian fur seal colony on Kanowna Island (39°10’S, 146°18’E) which has an annual pup production of *ca*3000 [27]. Data were collected as part of other concurrent studies on the foraging ecology of female Australian fur seals [29, 30]. During April-July of 2006–11, nursing females selected at random were captured using a modified hoop net (Fuhrman Diversified) and manually restrained until induction of isoflurane gas anaesthesia delivered via a portable vaporizer [31]. Once anaesthetized, individuals were removed from the hoop net and secured to a board before being weighed on a suspension scale (± 0.5 kg) and measured (± 0.5 cm) for axillary girth, flipper length (FL), standard length (SL) and axis length (nose to fore-flipper insertion point along dorsal mid-line). Individuals were then equipped with an electronic dive behaviour recorder (MK10, Wildlife Computers, Redmond, USA), a Fastloc GPS data logger (F1G 138A, Sirtrack, Havelock North, NZ) and a VHF transmitter (Sirtrack) glued in series to the pelage along the dorsal mid-line, just posterior to the scapula, using quick setting epoxy (Accumix 268, Huntsman Advanced Materials Pty). Together the devices represented <1% body mass and <1% cross sectional surface area and, hence, are unlikely to have negatively impacted the individual’s foraging behaviour [32]. Individuals were recaptured at the colony after a foraging trip to sea and the devices were removed by cutting the fur beneath them.

### Data processing and analyses

Dive behaviour records were analysed using the *diveMove* package [33] in R statistical environment (Version 2.12.2, [34]. Following zero-offset correction, and setting of a minimum dive-depth threshold of 5 m [30], dives were identified and characterised in terms of duration and maximum depth achieved. In addition to this, dives were classified into either benthic or pelagic using a custom written routine whereby individual dives were scored based on the proportion of time spent at the bottom of the dive multiplied by the maximum depth achieved during the dive. A kernel density estimate of the resulting score reveals a bimodal distribution, values to the left of the nadir between the two modes were taken to represent pelagic dives and values to the right of the nadir, benthic dives [35]. While the data from the GPS loggers were accurate, some highly erroneous locations still existed and, to remove these, data were filtered using a basic speed filter [36]. After filtering, GPS locations were linearly interpolated along each foraging track to be spaced evenly at 10 min intervals and merged with dive behaviour data [36] to provide a spatial location for each foraging (benthic) dive. Female Australian fur seals have previously been shown to be almost exclusively benthic foragers, conducting dives continuously while transiting (i.e. little surface travel) between focal foraging areas [29, 37]. Consequently, to investigate the potential influence of anthropogenic structures specifically on foraging behaviour, areas of intensive foraging activity were determined using first-passage diving analysis (FPD) following the methods of Hoskins et al. [35]. Briefly, FPD is a modification of first-passage time analysis [38] where the analysis substitutes time spent within an area for time spent underwater, for both the identification of the operational scale and final analysis steps (see [35] for further detail).

A presence-only model, MaxEnt [39], was then used to model the potential intensive foraging areas of each individual and to assess the relative importance of sea floor topography and anthropogenic structures to these areas. MaxEnt was chosen as it provides comparatively robust models when occurrence datasets are small (e.g. as low as 5 occurrence points) as is the case in this study (see [40]). Seven explanatory variables (depth, seafloor complexity, and Euclidean distances to colony, coast, pipeline/cables, wells and shipwrecks) were included in the models. Depth was determined from the 250 m grid cell resolution Australian Bathymetry and Topography Grid [41] and used to derive a complexity measure of local variability in benthic terrain [42], as variation in structural complexity has been observed to influence benthic fish and invertebrate communities [43]. The complexity measure refers to the second derivative or rate of change in the slope and is a measure of local variability in benthic terrain. Complexity was calculated based on a cell neighbourhood of 3 x 3. The location of wells, oil and gas pipelines, cable routes and shipwreck locations were obtained from Geosciences Australia, Heritage Victoria and the Department of Community, Planning and Development (Australia).

Euclidean distances to colony, coast, pipeline/cables, wells and shipwrecks were calculated using Spatial Analyst in ArcGIS 10 (ESRI, Redlands, USA) at a 250 m grid cell resolution in the study area encompassing Bass Strait. Collinearity (high correlation) between the seven explanatory variables in the model was investigated by estimating the variance inflation factor (VIF), using the *car* package in R, with an upper threshold value of three indicating collinearity. No collinearity was recorded and individual MaxEnt models were built by combining the seven explanatory variables with dive localities for each seal. Similar to Phillips and Elith [44], we partitioned the intensive foraging locality dataset into 70/30% for training and evaluation, respectively. It is important to note that if multiple intensive foraging localities were recorded in a single 250 m grid cell, these were combined to represent a single foraging event to avoid pseudo-replication in in the model. Default settings were used to build MaxEnt models; convergence threshold (0.00001), maximum iterations (1000), auto features, regularization multiplier (r = 1) and background points (10000). The 30% of dive localities set aside from model development were used to evaluate each model based on Area Under the Curve (AUC) using the MaxEnt GUI.

The proportion of the foraging trips associated with anthropogenic sea floor structures was investigated by determining from the complete GPS tracks the amount of time individuals were within a 250 m radius of an anthropogenic sea floor structure. A 250 m buffer was chosen to incorporate the potential locational errors in the anthropogenic structures. Furthermore, this buffer was also deemed relevant from a biological view point. While the influence of offshore artificial reefs on fish assemblages is considered localised (< 30 m), an important prey group for Australian fur seals (Family Monacanthidae, [24]) has been found to be associated with artificial reefs at a distance of up to 100 m [45]. Consequently, a buffer of 250 m was chosen to be conservative.

Generalized linear models (MuMin package in R, [46]) were then used to investigate relationships between individual morphological characteristics and the relative importance (as determined by MaxEnt models, arcsin transformed) of anthropogenic sea floor structures on intensive foraging locations and the (arcsin transformed) proportion of time spent foraging near them. Unless otherwise stated, date are presented as Mean ± SE and results considered significant at *P* < 0.05.

## Results

**Fig 2:**
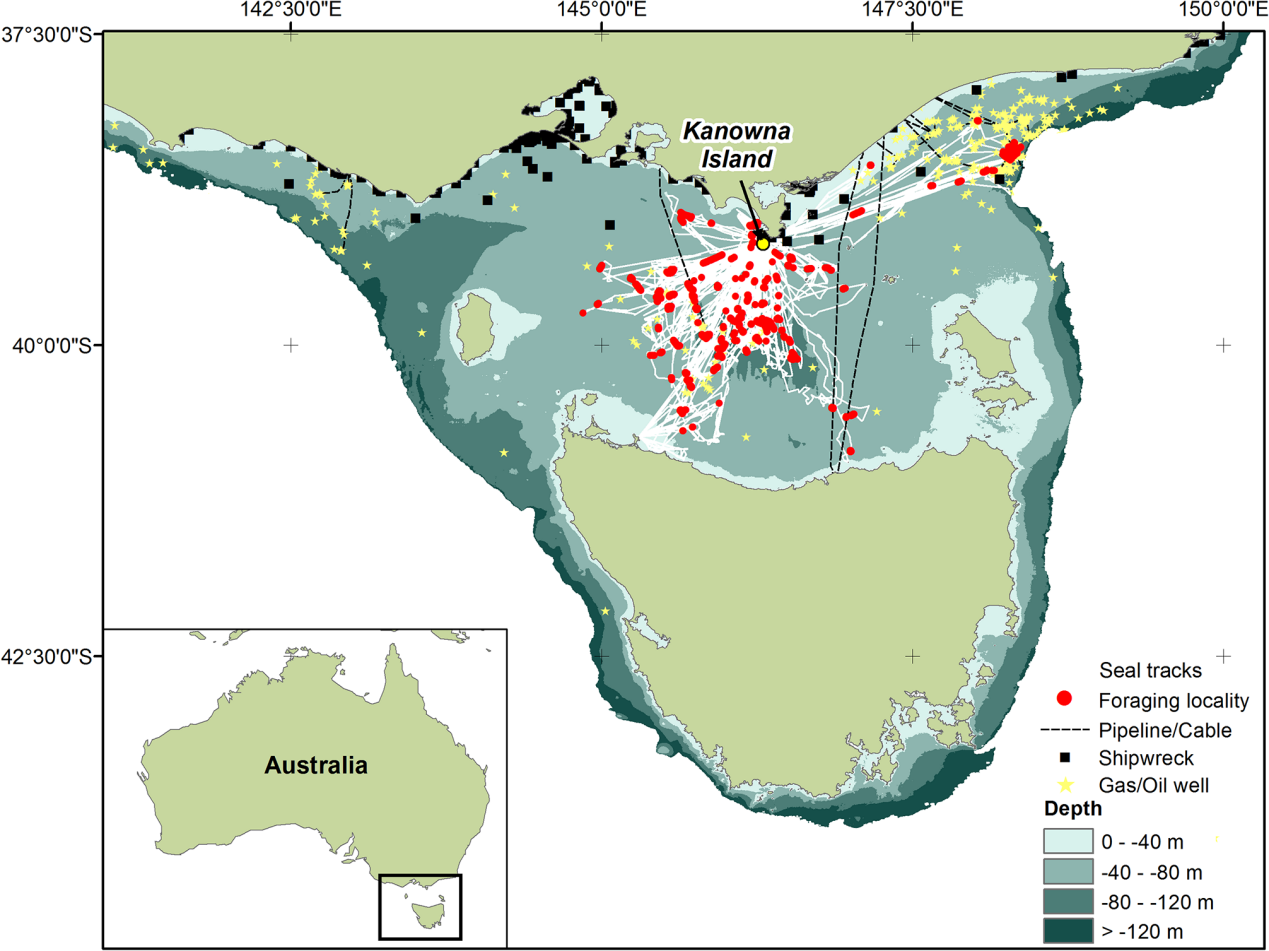
At-sea movements of adult female Australian fur seals from the Kanowna Island colony and the location of anthropogenic sea floor structures in Bass Strait, south-eastern Australia. Red circles indicate grid cells where intensive foraging activity occurred.

**Fig 3:**
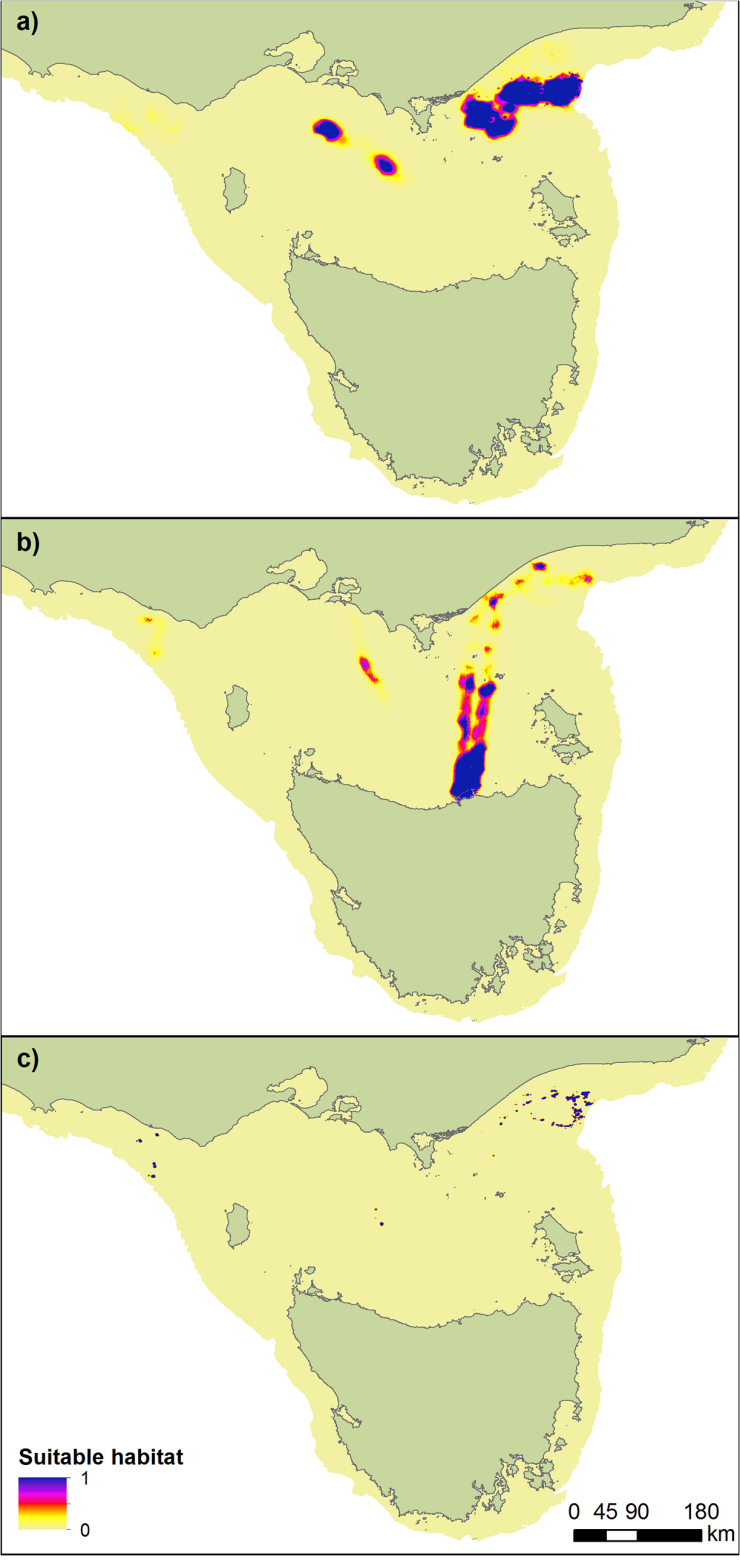
MaxEnt predictions of suitable foraging habitat for three of adult female Australian fur seals from the Kanowna Isl and colony. a) individualization towards shipwreck areas (individual 36); b) individualization towards pipeline/cable areas (individual 11); c) individualization towards oil well areas (individual 25).

A total of 36 individuals were recaptured after 1–8 foraging trips to sea (3–55 d). However, the majority (70%) of individuals were recaptured after a single trip and battery life limitations of the GPS logger resulted in only 5 individuals having full data sets for more than one complete trip. Hence, to remove the potential for bias from individuals with records of multiple foraging trips, only the first foraging trip (6.0 ± 0.6 d; 855 ± 111 dives) of each individual was used in further analyses. Their tracks (S1 File) and the locations of their intensive foraging areas were determined in relation to environmental and anthropogenic features (S2 File and Fig 2). All MaxEnt models returned AUC values > 0.90 suggesting strong performing models (Table 1). The MaxEnt models highlighted the individualisation of intensive foraging regions by the seals (Fig 3).

**Table 1:** Summary of individual characteristics of female Australian fur seals and the relative contribution of the explanatory variables used in MaxEnt models describing their foraging locations.

Seal	Mass	Length	Girth	Flipper*	Axis	Age	Relative contribution (%) in MaxEnt models^#^							Foraging	AUC
	(kg)	(cm)	(cm)	(cm)	(cm)	(y)	bathymetry	complexity	colony	coast	pipes/cables	wells	shipwrecks	cells^##^ (*n*)	
1	86.5	159.0	106.0	42.0	62.0	13	0.6	0.0	48.6	6.3	16.4	23.9	4.2	122	0.98
**2**	**91.5**	**165.0**	**110.5**	**43.5**	**72.5**	**12**	**4.5**	**0.3**	**6.6**	**37.1**	**16.2**	**25.8**	**9.5**	**14**	**0.99**
3	77.0	156.5	95.0	45.0	60.5	8	0.5	3.6	5.1	54.0	8.1	18.8	9.9	51	0.99
**4**	**82.0**	**161.5**	**105.0**	**47.5**	**65.0**	**8**	**0.7**	**0.1**	**18.8**	**13.2**	**31.4**	**22.4**	**13.5**	**57**	**0.98**
5	75.0	152.5	104.0	39.5	61.5	10	0.0	0.3	6.2	67.0	14.2	6.8	5.6	11	0.99
6	59.0	142.5	97.5	41.0	61.5	4	8.6	26.8	26.1	29.8	0.0	8.6	0.1	11	0.93
7	90.0	160.0	106.5	43.5	66.5	-	2.7	0.0	67.7	9.3	2.4	13.6	4.3	31	0.98
8	86.5	155.0	101.0	42.5	61.0	-	1.4	0.2	4.5	72.1	9.2	4.2	8.4	57	0.98
9	91.0	160.5	112.5	41.0	64.5	-	20.6	0.0	5.7	61.2	11.6	0.9	0.0	8	0.92
10	89.0	160.0	110.5	43.5	69.0	12	7.6	0.0	61.6	3.3	14.6	6.1	6.9	9	0.99
**11**	**84.0**	**157.0**	**106.0**	**48.5**	**64.5**	**-**	**0.0**	**0.3**	**0.0**	**5.8**	**58.1**	**6.1**	**29.7**	**29**	**0.95**
12	71.0	149.0	93.5	39.0	58.0	5	0.0	22.0	49.2	19.9	4.2	1.9	2.8	21	0.99
13	59.0	142.0	96.0	41.0	62.5	4	3.2	17.3	2.4	40.7	16.9	13.7	5.8	37	0.99
14	67.0	146.5	97.5	41.0	63.5	5	1.5	1.9	9.3	39.2	19.6	6.8	21.7	27	0.99
15	69.0	142.0	102.5	39.0	62.0	4	11.3	13.1	42.6	5.3	5.0	20.1	2.5	17	0.99
**16**	**81.0**	**151.5**	**103.0**	**42.5**	**64.0**	**6**	**0.2**	**0.0**	**6.0**	**20.3**	**25.6**	**47.6**	**0.4**	**193**	**0.99**
17	72.0	154.5	100.5	44.5	69.5	8	1.3	25.3	18.5	18.1	23.3	4.8	8.9	32	0.99
18	91.5	159.0	118.5	45.5	68.0	7	0.6	0.0	62.9	22.4	10.7	0.4	3.0	34	0.99
19	80.0	150.0	106.0	41.5	72.5	12	2.5	0.1	30.0	40.3	15.2	6.5	5.4	37	0.99
20	63.5	146.5	98.5	40.5	64.5	6	0.0	0.0	2.6	56.9	30.1	0.4	10.0	54	0.97
21	77.0	149.5	109.5	42.5	68.0	-	2.6	9.6	37.9	37.2	9.6	0.3	2.8	25	0.99
**22**	**81.5**	**156.5**	**112.0**	**43.5**	**66.5**	**10**	**1.0**	**0.0**	**16.5**	**27.9**	**53.9**	**0.7**	**0.0**	**15**	**0.99**
23	55.0	139.5	93.5	40.5	59.0	-	0.0	1.7	20.0	45.8	7.4	17.7	7.4	27	0.99
24^	86.5	160.0	116.0	43.0	68.5	11	-	-	-	-	-	-	-	-	-
**25**	**51.0**	**135.0**	**89.5**	**40.0**	**58.5**	**4**	**0.0**	**0.0**	**0.3**	**0.3**	**39.6**	**59.5**	**0.4**	**17**	**0.99**
26	56.0	139.0	95.5	41.5	65.5	8	2.0	1.0	79.0	9.9	6.6	0.0	1.5	29	0.99
27^	62.5	147.5	95.5	43.5	60.0	4	-	-	-	-	-	-	-	-	-
**28**	**101.0**	**154.5**	**119.5**	**44.0**	**73.0**	**-**	**2.0**	**0.2**	**28.1**	**4.8**	**8.3**	**36.7**	**20.0**	**15**	**0.95**
**29**	**71.0**	**144.5**	**98.0**	**41.5**	**64.0**	**7**	**1.3**	**0.3**	**0.7**	**43.0**	**18.3**	**16.1**	**20.3**	**80**	**0.90**
30	63.5	145.0	92.0	42.0	61.0	-	7.5	2.2	44.5	28.3	14.4	1.0	2.1	45	0.94
31	84.5	158.0	103.0	45.0	64.5	-	0.1	1.4	27.6	22.4	48.2	0.4	0.0	42	0.98
32	75.5	141.5	110.0	41.5	58.0	-	0.6	13.1	6.9	35.3	6.1	23.5	14.6	31	0.90
33	88.5	166.0	100.0	48.0	66.0	-	0.0	2.7	62.2	1.0	3.9	18.7	11.6	48	0.97
34	88.0	161.5	103.0	47.5	69.5	-	15.1	0.5	34.1	16.2	18.5	6.7	8.9	66	0.92
35	69.5	142.0	95.5	39.0	58.5	-	4.3	3.0	52.4	25.2	7.1	6.9	1.0	31	0.92
**36**	**87.5**	**159.0**	**104.5**	**42.5**	**66.5**	**-**	**0.0**	**0.0**	**3.9**	**20.8**	**22.4**	**19.7**	**33.1**	**35**	**0.94**
Mean	76.8	151.9	103.0	42.7	64.4	7.6	3.1	4.3	26.1	27.7	17.6	13.2	8.1	39.9	0.97
SE	2.1	1.4	1.3	0.4	0.7	0.6	0.8	1.3	4.0	3.4	2.5	2.4	1.4	6.1	0.01

Individuals in bold had distance to anthropogenic structure as the greatest contributing factor in models.

*fore-flipper length.

^#^bathymetry and sea floor complexity at location of dives and their distance to all other features

^insufficient dive locations to build MaxEnt model.

^##^grid cells containing areas of intensive foraging activity.

Interrogation of the relative importance of the explanatory variables from the MaxEnt models indicated that, on average, the greatest contribution to the models were distance to colony and coast while bathymetry and complexity accounted for the least amount of variation overall. However, for 20 (55%) individuals, distance to anthropogenic sea floor structures accounted for >30% of variance in the location of intensive foraging activity and for 9 (25%) individuals it contributed the greatest proportion of the variance (Table 1). There was no significant difference between years in the contribution of distance to anthropogenic sea floor structures to the MaxEnt models (Kruskal-Wallis *H*_5,34_ = 3.27, *P* > 0.5). Further interrogation of the relative importance of distance to anthropogenic structure variables suggested that the type of structure (i.e. pipeline/cable route, oil/gas wells, shipwreck,) differed in mean influence (18%, 13% and 8%, respectively; Kruskal-Wallis *H*_2,102_ = 10.46, *P* < 0.01).

A total of 26 (72%) individuals were observed spending time in the vicinity of anthropogenic sea floor structures. Of the animals spending time in the vicinity of anthropogenic structures, 96% visited pipelines and cable routes, 42% visited oil/gas wells and 23% visited shipwrecks. However, the proportion of foraging trips animals spent near anthropogenic structures varied greatly, from <1% for 20 individuals that visited structures briefly en route to other areas, to >20% for 4 seals (up to 76% in one individual) that concentrated foraging effort in the vicinity of pipelines/cables and wells. Greater than a third (35%) of individuals visited more than one type of anthropogenic sea floor structure.

Models explaining the relationships between individual morphological characteristics and the relative importance of anthropogenic structures in foraging locations indicated that the most parsimonious models included the FL/SL ratio, Mass and the Axis/SL ratio (Table 2). Due to the lack of a single best model, model averaging was conducted and the FL/SL ratio was found to have a weak positive influence on the relative importance of anthropogenic structures on seal foraging location. None of the measured individual morphological characteristics were found to influence the proportion of time spent in the vicinity of structures. Similarly, there were no relationships observed between the proportion of time spent in the vicinity of structures and the overall foraging effort (dive rate, trip duration or maximum distance travelled) of individuals

**Table 2:** Comparison of linear models for individual morphological characteristics explaining the relative contribution in MaxEnt models of foraging locations associated with anthropogenic sea floor structures.

Model	Df	∆AICc	AIC weight	R^2^
FL/SL + Intercept	3	0.00	0.409	0.13
FL/SL + Mass + Intercept	4	1.75	0.171	0.15
Intercept	2	2.11	0.142	-
FL/SL + Axis/SL + Intercept	4	2.26	0.132	0.13
FL/SL + Mass + Axis/SL + Intercept	5	4.29	0.048	0.15

## Discussion

The Australian fur seal population is still recovering from past exploitation [27] and the Kanowna Island colony has been growing slowly at approximately 2% per annum since the late 1990s ([27], Arnould *unpublished data*). In addition, the duration of foraging trips in the present study was within the range (3–7 d), and the diving behaviour consistent with that, previously reported for individuals from this colony [29, 35, 37]. Furthermore, the availability of anthropogenic structures within the foraging range of individuals from the study colony did not change during the sampling period. Hence, results of the present study are considered to reflect normal foraging behaviour in relation to environmental and anthropogenic influences for individuals from this colony.

Distance to anthropogenic structures accounted for a substantial proportion (>30%) of the variance in intensive foraging area locations for over half the individuals in the present study. While these results do not indicate direct specific use of such structures as forage sites, they suggest a spatial link between the presence of structures and potential foraging habitat. As artificial reefs increase habitat connectivity for invertebrate and fish species [13, 14], their influence on foraging habitat for predators such as Australian fur seals may extend beyond their immediate location. Indeed, the individual instrumented with a video data logger (Fig 1), in addition to searching for prey along the pipeline itself, was observed to forage repeatedly on benthic fish (gurnards, Family Triglidae) at an estimated distance of up to 50 m from the pipeline on the leeward side to the prevailing currents (as evidenced from sand accumulation). Increased vertical relief from anthropogenic structures may influence micro-habitat structure for benthic prey species over a wide area by changing local currents and nutrient transport [12, 47]. Consequently, as Bass Strait has a mostly uniform bathymetry with few benthic features [41], the ecological impact of anthropogenic structures, and their benefit to Australian fur seals, may be more widespread than just at the structures themselves.

Interestingly, pipelines and cable (electricity and telephone) routes were the most visited and most influential structures associated with foraging locations despite such features having limited vertical scope and habitat complexity (and, thus, diversity in prey habitat) in comparison to wells and shipwrecks. However, pipelines/cable routes may represent greater overall area and provide habitat connectivity for prey species [13] potentially making them more profitable sites to exploit. Furthermore, the pipeline shown in Fig 1 had only been installed 5 years earlier, indicating the potentially rapid development of such structures as important foraging sites. Similar rapid use of such structures has been reported for grey and common seals in European waters [18].

There was substantial variation in the amount of time individuals spent at anthropogenic structures (<1% to >75%). Individuals that briefly visited anthropogenic structures, some at multiple locations, did so throughout the course of their foraging trip. This potentially reflects the many small, localised (e.g. capped oil/gas wells, shipwrecks) or narrow (pipelines/cables) anthropogenic structures distributed within Bass Strait (Fig 2), that these structures provide limited benefits and/or that some individuals were simply passing these structures while accessing foraging locations not associated with them. In contrast, all 4 individuals that spent >20% of their time at sea near anthropogenic structures travelled 65–175 km in comparatively direct routes to do so suggesting prior experience at these sites. These findings suggest such sea floor structures may act as important *de facto* artificial reefs for some individuals in an otherwise seemingly barren benthic seascape [29]. Indeed, video data from animal-borne cameras have revealed several individuals (>4) at the same time foraging in the vicinity of such structures. A similar frequency of visitation to wind turbines and pipelines has recently been observed in grey (*Halichoerus grypus*) and common (*Phoca vitulina*) seals [18].

The present study only followed individuals for a single foraging trip such that it is not possible to determine whether the level of visitation or importance of anthropogenic structures to individual Australian fur seals is consistent through time, as has been observed in grey and common seals [18]. However, recent studies investigating intra- and inter-individual variation in diet (assessed through stable isotope analysis of whiskers) have revealed a high level of consistency in individual foraging strategies of female Australian fur seals (Kernaleguen et al. *unpublished data*). Hence, the results of the present study may reflect the level of long-term association with anthropogenic sea floor structures by Australian fur seals. Further tracking of at-sea movements by individuals over multiple foraging trips is needed to confirm this.

Age and size (mass, length), a correlate of age [48], were found not to influence the relative importance of anthropogenic sea floor structures in the MaxEnt models which suggests that use of such anthropogenic features are not a consequence of experience. However, the FL/SL ratio, a factor which can affect manoeuvrability [49] was found to be weakly influential. This index has also been found to influence diet [50] and its importance may reflect morphological advantages in chasing particular prey, perhaps those associated with artificial and natural reef structures or the regions around them.

The increasing demand for energy resources around the world has led to the development of many offshore structures for oil and gas extraction as well as wind and tidal power generation. Whereas much concern has been raised about the potential negative environmental impacts of such developments [51–53], the results of the present study and those of Russell et al. [18] highlight potentially beneficial outcomes. Many of these developments are situated in regions where populations of benthic foraging pinnipeds are still recovering slowly from past exploitation or are declining (e.g. common, grey, Mediterranean monk *Monachus schauinslandi*, southern sea lion *Otaria flavescens* [19]. In addition to such structures becoming habitat for fish communities [14, 54], they could represent important additional foraging sites for pinniped species whose habitats have been degraded by decades of commercial fishing activity [22]. Consequently, if managed appropriately, marine industrial development may enhance the prospects of these coastally restricted populations. However, further research is needed to specifically determine the benefits, in terms of foraging success, to individuals that frequent artificial reefs in order to properly assess such prospects.

## Supporting Information

S1 File This file contains the GPS track files of all individuals used in study. (ZIP)

S2 File This file contains the foraging dive locations with the explanatory variables extracted for each dive location. (XLSX)
